# Use of Open Surface Plasmon Resonance (OpenSPR) to Characterize the Binding Affinity of Protein–Protein Interactions

**DOI:** 10.21769/BioProtoc.4795

**Published:** 2023-09-05

**Authors:** Cassie Shu Zhu, Jianhua Li, Haichao Wang

**Affiliations:** 1 The Feinstein Institutes for Medical Research, Northwell Health, 350 Community Drive, Manhasset, NY, USA; 2 Donald and Barbara Zucker School of Medicine at Hofstra/Northwell, 500 Hofstra Blvd., Hempstead, NY, USA

**Keywords:** Procathepsin L (pCTS-L), Toll-like Receptor 4 (TLR4), Receptor for Advanced Glycation End Products (RAGE), OpenSPR, NTA Sensor Chip

## Abstract

Surface Plasmon Resonance(SPR) is a label-free optical technique to assess protein–protein interaction kinetics and affinities in a real-time setting. Traditionally, Biacore SPR employs a continuous film of gold to detect any change in the angle of re-emitted light when the refractive index of a ligand conjugated to the flat gold surface is altered by its interaction with a local analyte. In contrast, the Nicoya Lifesciences’ OpenSPR technology uses gold nanoparticles to detect small changes in the absorbance peak wavelength of a conjugated ligand after its engagement by an analyte. Specifically, when broadband white light is shone onto the gold nanoparticles, it produces a strong resonance absorbance peak corresponding to the refractive index of a ligand conjugated to the surface of gold nanoparticles. Upon its interaction with an analyte, however, the absorbance wavelength peak of the conjugated ligand will be changed and timely recorded as sensorgrams of dynamic ligand–analyte interactions. Thus, the improvement in the detection method (from traditional detection of changes in the angle of re-emitted light to the contemporary detection of changes in the wavelength of the absorbance peak) features OpenSPR as a cost-effective and user-friendly technique for in-depth characterization of protein–protein interactions. Here, we describe the detailed method that we used to characterize procathepsin L (pCTS-L) interactions with two putative pattern recognition receptors (TLR4 and RAGE) using the 1st generation of Nicoya Lifesciences’ OpenSPR instrument with a 1-channel detection.

Key features

• Nicoya OpenSPR is a benchtop small-size equipment that provides in-depth label-free binding kinetics and affinity measurement for protein–protein interactions in real-time fashion.

• This technology is relatively intuitive and user-friendly for scientists at any skill level.

• OpenSPR sensors employ nanotechnology to reduce the cost of manufacturing complex optical hardware and Sensor Chips, and similarly reduce the consumption of precious analyte samples.

• The manufacturer provides online training for OpenSPR (Catalog: TRAIN-REMOTE) and TraceDrawer (Catalog: TRAIN-TD) to customer scientists.

## Background

In order to understand complex mechanisms underlying the regulation of various biological processes, it is often essential to characterize protein–protein interactions under many physiological or pathological conditions. For many years, surface plasmon resonance (SPR) has been widely used to analyze all types of interactions between protein–protein or protein and other molecules such as nucleic acids, lipids, and carbohydrates (Willander and Al-Hilli, 2009;Drescher et al., 2018). Traditionally, Biacore SPR uses a continuous film of gold to detect changes in the angle of re-emitted light when the refractive index of a ligand conjugated to the flat gold surface is altered by its interaction with a local analyte (Willander and Al-Hilli, 2009;Drescher et al., 2018). In contrast, the newly developed Nicoya Lifesciences’ OpenSPR employs gold nanoparticles to detect small changes in the absorbance peak wavelength of a conjugated ligand after its binding to an analyte. Nevertheless, both traditional SPR and contemporary OpenSPR can give real-time information about the binding kinetics and affinities of dynamic interactions between various molecules.

During the last few years, we have used Nicoya Lifesciences’ first generation 1-channel OpenSPR to determine the binding affinities between antigens and antibodies (Chen et al., 2020) or cytokines and putative receptors (Qiang et al., 2022;Zhu et al., 2023). Briefly, a ligand protein is immobilized onto nitrilotriacetic acid (NTA)-conjugated gold nanoparticles in a specific orientation, and an analyte solution is subsequently injected at several increasing concentrations into the microflow system. When broadband white light is shone onto the gold nanoparticles, it produces a strong resonance absorbance peak that is specific to the refractive index of a local ligand conjugated to the gold nanoparticles. If an analyte binds the ligand, it will induce a change in the wavelength of the absorbance peak of the ligand, which can be recorded in the sensorgram as an increase in the SPR signal. After a desired association time, a solution without the analyte is injected to dissociate the complex between ligand and analyte. If the analyte dissociates from the ligand, a decrease in SPR signal would be observed. By dividing the dissociation rate (*k_off_*) by the association rate (*k_on_*), the equilibrium dissociation constant (*K*_D_) can be calculated as a measure of the affinity between these specific ligand–analyte interactants. Thus, the improvement in the detection method (from traditional detection of changes in the angle of re-emitted light to the contemporary detection of changes in the wavelength of the absorbance peak) features OpenSPR as a cost-effective and user-friendly technique for in-depth characterization of protein–protein interactions. Here, we describe the detailed method that we used to characterize procathepsin L (pCTS-L) interaction with two putative pattern recognition receptors (TLR4 and RAGE) using Nicoya Lifesciences’ 1^st^generation OpenSPR instrument with a 1-channel detection (Zhu et al., 2023).

## Materials and reagents

**Reagents**

1. Extracellular domain of human TLR4 (residue 1-631, 70.5 kDa) (Sino Biological, catalog number: 10146-H08B)
2. Extracellular domain of human RAGE (residue 1-344, 36.0 kDa) (Sino Biological, catalog number: 11629-H08H)
3. Human pCTS-L corresponding to residue 17-333 of respective procathepsin L carrying a N-terminal 6× His tag was expressed in*E. coli*BL21 (DE3) pLysS cells and purified as previously described (Zhu et al., 2023).

**Solutions**

1. Phosphate buffered saline (PBS) containing 10 mM Na_2_HPO_4_, 10 mM NaH_2_PO_4_, 140 mM NaCl, and 3 mM KCl at pH 7.4 (Nicoya Lifesciences, Kitchener, catalog number: NI-PBS)
2. HBS-T running buffer containing 0.01 M HEPES, 0.15 M NaCl, and 0.005% Tween-20 at pH 7.4 (Nicoya Lifesciences, catalog number: HBS-T)
3. NiCl_2_solution (40 mM, store at 2–8 °C) and imidazole solution (200 mM, store at 2–8 °C) prepared using the NTA Reagent Kit (Nicoya Lifesciences, catalog number: NTA-RK)
4. Deionized water filtered through 0.2 μm vacuum filter bottle system (Corning, catalog number: 431097)
5. 80% isopropanol (v/v in deionized water, prepared from isopropanol) (Sigma-Aldrich, catalog number: 563935)

**Laboratory supplies**

1. Buffer bottles (Nicoya Lifesciences, catalog number: BTL-SQ-250)
2. Tweezers (included in the standard supplies of OpenSPR equipment)
3. Lint-free wipes (Fisher Scientific, catalog number: S47299)
4. Disposable syringes (Nicoya Lifesciences, catalog number: SYR-PL-50)
5. Gastight glass syringes (Nicoya Lifesciences, catalog number: SYR-G)
6. Blunt-end injection tips (Nicoya Lifesciences, catalog number: TIP-BLUNT-50)
7. OpenSPR Nitrilotriacetic Acid (NTA) Sensor Chip (Nicoya Lifesciences, catalog number: SEN-Au-100-10-NTA)
8. NTA Reagent Kit for making 40 mM NiCl_2_solution and 200 mM imidazole solution (Nicoya Lifesciences, catalog number: NTA-RK)
9. Nitrile or Latex gloves
10. Declogging Kit (Nicoya Lifesciences, catalog number: DECLOG OpenSPR)

## Equipment

1. Nicoya Lifesciences’ 1^st^ (OpenSPR) 1-Channel Instrument (Kitchener, catalog number: REV 3.0,Figure 1).
  **Figure 1.**
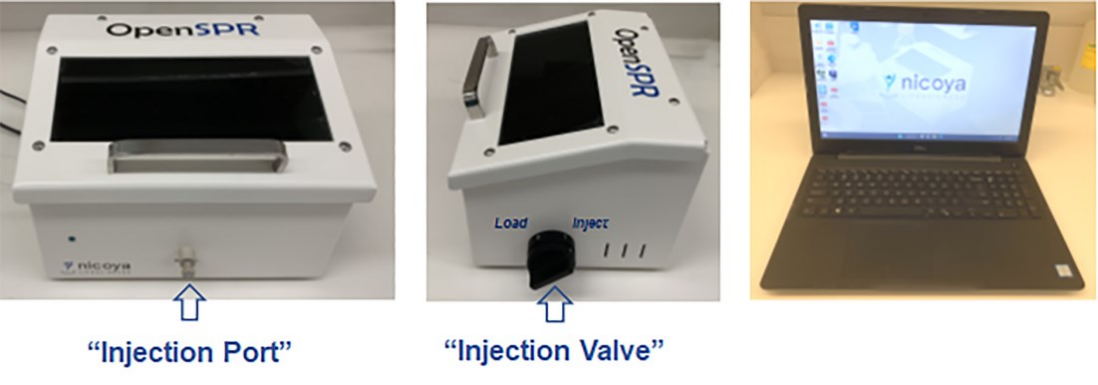
Photos of an OpenSPR 1-Channel Instrument and a laptop computer with installed Nicoya Lifesciences software
  *Note: The OpenSPR is operated by the user manually, using a laptop computer with an Experiment Data Interface that allows the user to control various aspects of the OpenSPR instrument, such as changing running buffer, fluid flow rate, as well as sample injection. For instance, when a sample is manually injected into the*Sample Loop*via the Injection Port, the user then needs to switch the Injection Valve clockwise to the*Inject*position to initiate the delivery of the injected samples from the*Sample Loop*to the sensor.*
2. Laptop computer with installed Nicoya Lifesciences software (Experiment Data Interface) for control of the instrument and fast real-time data acquisition (Figure 1).

## Software and datasets

1. TraceDrawer Kinetic Data Analysis Software V.1.6.1 (Nicoya Lifesciences) for data processing and analysis of kinetic and affinity constants.

## Procedure

1. Set up the OpenSPR instrument and software according to the manufacturer’s detailed instructions in the OpenSPR manual, which includes the connection of the OpenSPR instrument to the computer with a USB cable and the fluidic setup of three bottles (for buffers and waste).
2. Open the Experiment Data Interface on the computer, and click *Start* to proceed to the preparation of the instrument by:
  - Indicating your running buffer and priming the fluidics of the OpenSPR by filling the pumps and tubing with the indicated running buffers (HBS-T, pH 7.4) at a flow rate of 150 μL/min for 2.5 min to clear out any contamination or bubbles out of the fluidic lines prior to your test.
  - Taking a new optical reference spectrum without a Sensor Chip in the instrument to calculate the sensor absorbance signal before loading a new Sensor Chip to start a new test.
  - Loading a new NTA Sensor Chip prior to the start of a test:
    i. Clean the face of the Flow Cell with a lint-free wipe soaked in 80% isopropanol and allow it to air dry completely before installing a new Sensor Chip.
    ii. Rinse the Sensor Chip thoroughly with distilled water and allow it to air dry completely to prevent leaking and bubble formation in the Flow Cell.
    iii. Carefully slide the Sensor Chip into the Sensor Holder in the correct orientation and place the Sensor Holder into the instrument by first aligning it with the Fluidics Block, then slowly bring it into contact.
3. Prepare the test by:
  - Switching the Injection Valve to the *Load* position and preparing for sample injection by:
    i. Slowly rinsing the Injection Port and Sample Loop with at least 1.0 mL of running buffer (HBS-T, pH 7.4) to flush any previous sample/solution out of the Sample Loop fluidic lines.
    ii. Purging the Sample Loop using the same buffer syringe filled with at least 1.0 mL of air to push the air through the Sample Loop, to remove excess buffer in the loop via the outlet line.
  - Removing bubbles within the fluidic lines.
    i. Set the pump speed at 150 μL/min and turn the Injection Valve counterclockwise to the*Load*position.
    ii. Use a syringe with a blunt-end tip to inject 300 μL of 80% isopropanol via the Inject Port into the Sample Loop, and then turn the Inject Valve clockwise to the *Inject* position to deliver isopropanol to the sensor.
    iii. Turn the Injection Valve counterclockwise back to the*Load*position.
4. Immobilize the 6× His Tag-containing pCTS-L ligand onto the NTA sensor according to instructions.
  - Surface conditioning: set the pump speed to 150 μL/mL and use disposable syringes to fill the*100 μL*Sample Loop with 200 μL of imidazole solution (200 mM), and then turn the Injection Valve to the *Inject* position to initiate delivery of the injected imidazole solution from the*Sample Loop*to the sensor to clean the sensor surface. After 5 min, turn the Injection Valve counterclockwise back to the *Load* position.
  - Rinse the Inject Port and Sample Loop with at least 1.0 mL of running buffer (HBS-T, pH 7.4) and purge with at least 1.0 mL of air as described in step 3a before the next injection.
  - Surface activation: set the pump speed to 20 μL/mL and use disposable syringes to fill the Sample Loop with 200 μL of NiCl _2_ solution (40 mM), and then switch the Injection Valve to the *Inject* position to initiate delivery of the injected NiCl_2_solution from the *Sample Loop* to the sensor to activate the NTA sensor surface. Leave the Inject Valve in the *Inject* position for the entire duration of the activation time until a stable baseline is obtained, and then turn the Injection Valve counterclockwise back to the *Load* position.
  - Rinse the Inject Port and Sample Loop with at least 1.0 mL of running buffer (HBS-T, pH 7.4), and purge with at least 1.0 mL of air as described in step 3a.
  - Switch the running buffer from HBS-T to 1× PBS by:
    i. Clicking the *Stop* button in the Pump Menu of the Experiment Data Interface to stop the pump.
    ii. Switching the inlet tubing from the bottle containing HBS-T to the bottle containing 1× PBS (pH 7.4).
    iii. Resuming the pump by clicking the *Start* button in the Pump Menu.
  - Set the pump speed to 20 μL/mL and use disposable syringes with gastight blunt-end tips (TIP-BLUNT-50) to inject 200 μL of pCTS-L solution diluted in the running buffer (1× PBS, pH 7.4) to a final concentration of 50 μg/mL. Quickly turn the Injection Valve clockwise to the *Inject* position to initiate the delivery of the injected pCTS-L ligand solution from the *Sample Loop* to the NTA sensor. Leave the Injection Valve on the *Inject* position for the entire duration of the ligand interaction with the sensor surface. A representative sensorgram for immobilizing pCTS-L ligand onto an NTA Sensor Chip is shown below (Figure 2).
    **Figure 2.**
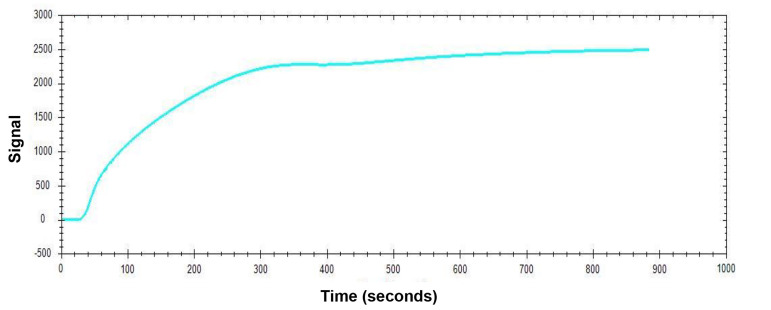
Sensorgram of pCTS-L Ligand Immobilization
*Note: For most protein ligands, the recommended ligand concentrations for immobilization are typically 10–50 μg/mL. We chose a higher concentration (50 μg/mL) of pCTS-L ligand to ensure its maximal immobilization to most active sites of the NTA sensor. The amount of ligand immobilized onto the NTA Sensor Chip can be determined by the subsequent increase in the response signal in corresponding sensorgrams. Once the immobilization signal has reached plateau levels, it indicates a state of maximal ligand immobilization. However, the user can repeat the immobilization procedure one more time to ensure there will be no additional further increase in the response signal (i.e., the “net signal shift”) corresponding to additional ligand immobilization, so that most (if not all) NTA open sites are occupied by the ligand to prevent non-specific interaction with analytes containing exposed histidine residues.*
  - When an injection is made, an Injection Details box will appear for the user to input the ligand name (pCTS-L) as well as the concentration (50 μg/mL), which will be used to identify the injection in the data analysis.
    *Note: For all injections, other than the Bubble Removal Injection and Analyte Injections, leave the Injection Valve in the *Inject* position for at least the entire duration of the interaction time of your sample with the sensor surface. When ready to perform the next injection, turn the Injection Valve back to the *Load* position, and rinse the Injection Port and Sample Loop with running buffer and purge the Sample Loop with air as described in step 3a.*
5. Once the immobilization signal has reached plateau levels, it indicates a state of maximal ligand immobilization. Allow the instrument to stabilize with buffer running until the slope of the baseline is < 5 pM/min, which may take anywhere from 5 min to 1 h.
6. Rinse the Inject Port and Sample Loop with running buffer (1× PBS, pH 7.4), and purge with air as described in step 3a.
7. Prepare TLR4 analyte solution in the analysis running buffer (1× PBS, pH 7.4) at 3-fold serial dilutions (i.e., 283.7 nM, 94.6 nM, and 31.5 nM), and inject at least 200 μL of analyte solution at a flow rate of 20 μL/min in the order of increasing concentrations with an association time of 240 s and a dissociation time of 480 s.
  - Load syringe with 200 μL of TLR4 analyte solution at a concentration of 31.5 nM, insert it fully into the Injection Port, and then slowly depress the plunger to introduce the analyte into the instrument. Wait at least 5 s to stabilize the syringe fluidic pressure in the Sample Loop before removing the syringe from the Injection Port.
  - Turn the Injection Valve clockwise to the *Inject* position to initiate the delivery of the injected TLR4 analyte solution from the *Sample Loop* to the NTA sensor and wait for 240 s to ensure maximal interaction between ligand and analyte.
  - When the *Injection Remaining* counter reaches 00:00:00, quickly turn the Injection Valve back to the *Load* position, and wait for 480 s to allow maximal dissociation between ligand and analyte.
  - When the injection is complete and the baseline has settled, the user can move on to the next round of injection of TLR4 analyte at 94.6 nM and 283.7 nM, respectively, by repeating steps 6 and 7.
*Note: When an injection is made, an Injection Details box will appear to let the user input the analyte sample name (e.g., TLR4) as well as the concentration (e.g., 31.5 nM), so the software can identify this injection in subsequent data analysis. Before each analyte injection, the Injection Port and Sample Loop must be rinsed with analysis running buffer (e.g., 1× PBS, pH 7.4) and purged with air as described in step 3a to prevent cross-contamination between injections.*
8. To complete an experiment, click *Finish* to stop tracking the absorbance peak and create the final data files.
9. For the characterization of the pCTS-L-RAGE interaction, load a new NTA Sensor Chip and prepare the solution of RAGE analyte in the analysis running buffer (1× PBS, pH 7.4) at 3-fold serial dilutions (i.e., 250 nM, 83.3 nM, and 27.7 nM). Respectively, inject the *Sample Loop* with 200 μL of RAGE analyte solution at a flow rate of 20 μL/min in the order of increasing concentrations with an association time of 240 s and a dissociation time of 480 s following similar procedures as described in steps 2–8.
10. Data Analysis: Once the user finishes the test, the software will create several types of data files that will always be saved under Documents\OpenSPR\TestResults\ along with the name given by the user at the beginning of the test.
  - Open TraceDrawer Software and click *Add run* to select and import saved files and relevant curves (i.e., TLR4 or RAGE) for analysis.
  - Create an overlay by clicking *New Overlay* and then left click and drag a run to an overlay to perform data modifications in the Overlay windows.
  - On the left side of the screen, click *New Evaluation* to set the Evaluation Type to Kinetics Evaluation.
  - Define two timepoints where a concentration change occurs in the data, i.e., the beginning and end of the association phase.
  - In the Kinetics Evaluation table that displays a row with the selected timepoint, define the concentrations of each curve, and press *Next*.
  - In Fit model dropdown list, choose OneToOne model to initiate a 1:1 diffusion-corrected binding model with global fitting, then press Fit.
  - For more comprehensive instructions on the TraceDrawer Software, please refer to the manual.
*Note: The user can zero the graph by clearing all the data points from the graph and normalizing the response tracking to zero on the y-axis at any stage in the experiment where a new baseline is established. For instance, the user can zero the graph to make a relative comparison for subsequent injections at any stage after the ligand immobilization step and before injection of the analyte for analysis. The user can also open advanced settings to manually adjust the axes scale by clicking the gear (settings) button in the graph menu and setting custom axes before saving an image of the graph.*
11. Shutdown Procedure
  If the user does not plan to use the instrument in the near future, it is recommended to perform a full shutdown before rinsing all fluidic lines thoroughly with at least 20 mL of deionized water and 20 mL of 80% isopropanol at the maximum speed (150 μL/min), followed by purging the lines with a sufficient amount of air to dry out the tubing lines. For information regarding other rinsing procedures and maintenance, please refer to the manual of the instrument.

## Data analysis

**Figure 3.**
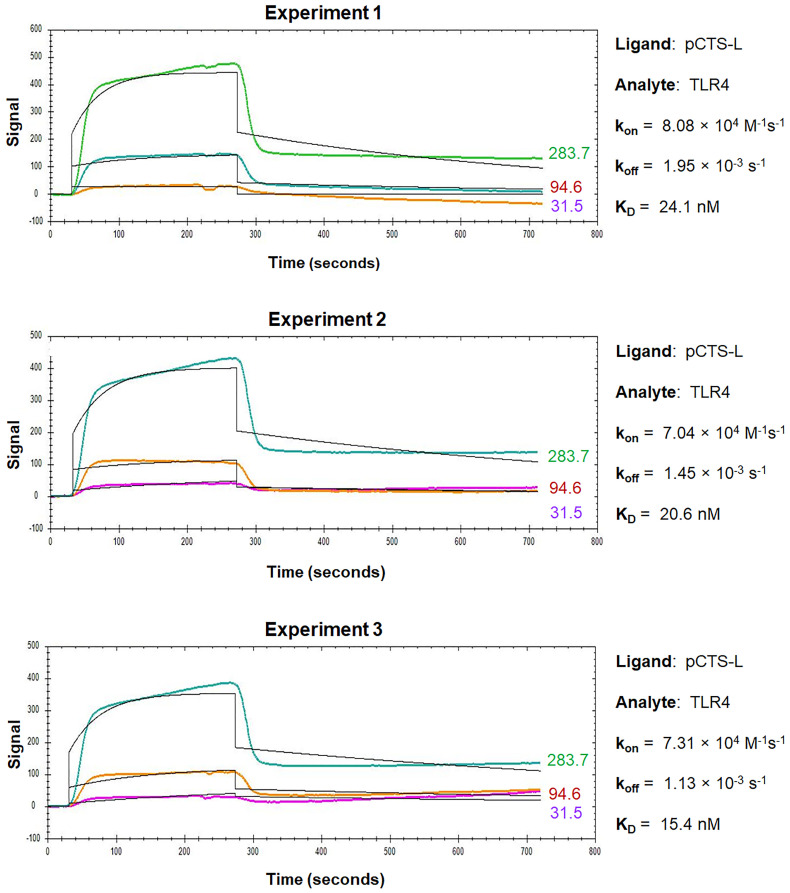
Sensorgrams of pCTS-L–TLR4 interactions. TLR4 was injected for a contact time of 240 s at increasing concentrations of 31.5 nM, 94.6 nM, and 283 nM, and dissociation was monitored for 480 s. The 1:1 models fit to the raw data are shown as solid black lines.

For the OpenSPR analysis of pCTS-L interactions with TLR4 or RAGE, pCTS-L is immobilized as a ligand on a NTA sensor, and TLR4 or RAGE are applied as an analyte at increasing concentrations. The dynamic changes of signal response are recorded in the sensorgrams, which include a baseline phase of the buffer response, an association phase during which the analyte is passed over the immobilized ligand, and a dissociation phase where buffer flow resumes, and the bound analyte is removed from the sensor surface (Figures 3and4).

**Figure 4.**
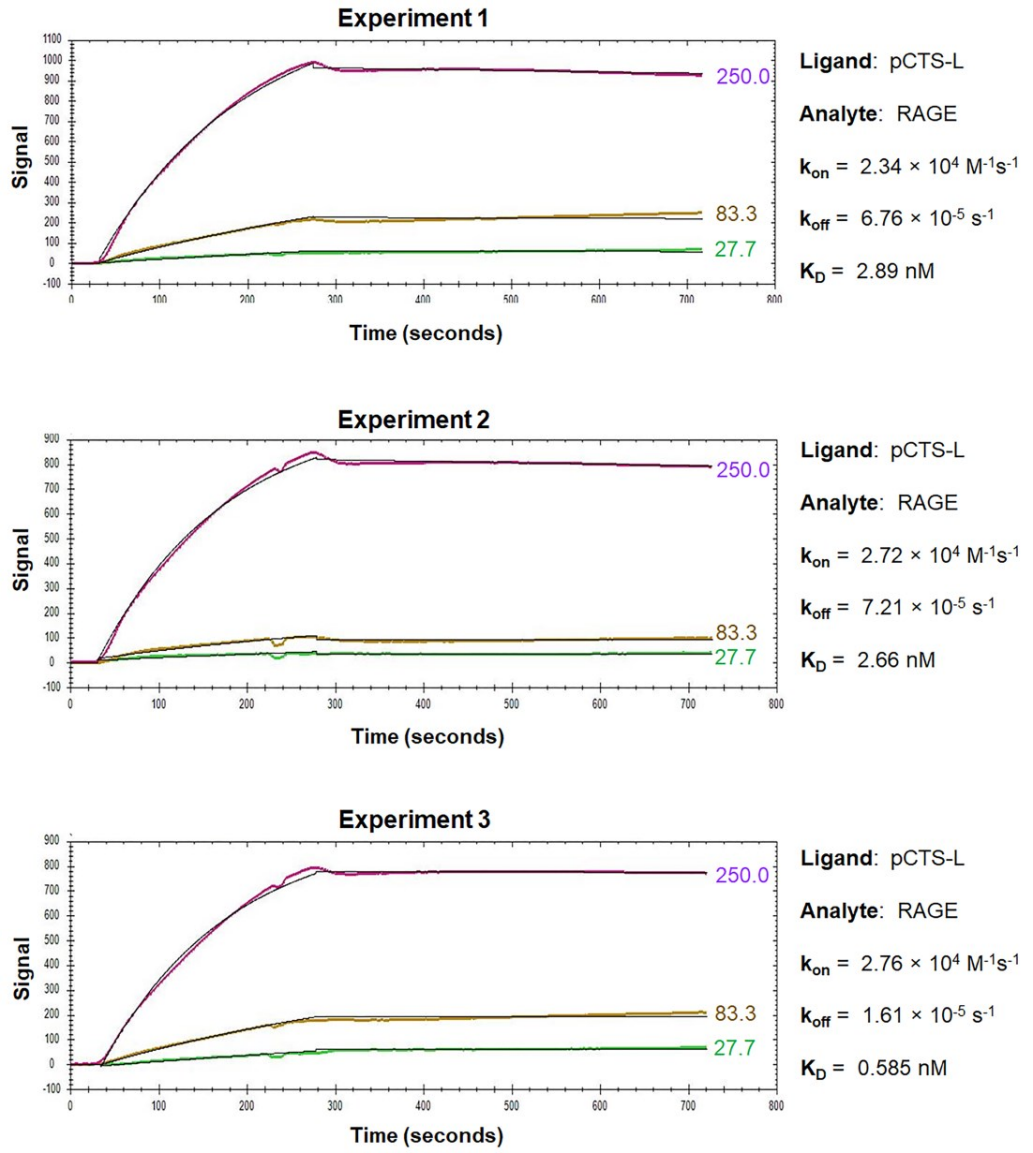
Sensorgrams of pCTS-L–RAGE interactions. RAGE was injected for a contact time of 240 s at increasing concentrations of 27.7 nM, 83.3 nM, and 250 nM, and dissociation was monitored for 480 s. The 1:1 models fit to the raw data are shown as solid black lines.

During the association phase, the sensorgrams reveal dynamic changes in the absorbance peak wavelength between the running buffer and the analyte in proportion to the analyte’s concentrations, and always exhibit some type of curvature as an indicator of exponential increase (Figures 3and4). When the sensorgram curve levels out, it indicates a state of equilibrium when the number of associations equals the number of dissociations. However, when the analyte is injected at a higher concentration, a new equilibrium is reached with a higher response (Figures 3and4), until all the ligand binding sites are occupied, and the maximal response is reached. Therefore, the concentration range of the injected analyte is important, because higher concentrations of analyte tend to produce curves in the upper part of the sensorgram, whereas lower concentrations will give low responses and little curvature. It is recommended to inject an analyte at 3–5 concentrations at a serial dilution in the range of 0.1–10-fold of the “equilibrium dissociation constant” (*K*_D_, i.e., 0.1 × *K*_D_ to 10 × *K*_D_), which will space the curves evenly over the sensorgram with both low and high responses. When the analyte injection ends, it will enter a dissociation phase, during which the curve of dissociation follows a single exponential decrease (Figure 3) but can sometimes be almost horizontal in the case of a strong interaction (Figure 4). To analyze a slow dissociation, the dissociation period should be long enough to have at least 5% signal decrease compared with the initial response.

OpenSPR experiments can be repeated multiple times to get an average *k_on_, k_off_*, and *K*_D_to increase reliability and reproducibility. Representative sensorgrams for kinetic interactions between pCTS-L and TLR4 or RAGE are shown in Figure 3 and Figure 4, respectively. The overall shape of the sensorgram curves is determined by the analyte concentration, association rate (*k_on_*), and dissociation rate (*k_off_*) constants between a particular pair of ligand and analyte. Within a particular ligand–analyte pair, the overall shape of the sensorgram curves was extremely similar between repeating experiments (Figures 3 and 4), indicating some degree of reproducibility and reliability of the OpenSPR technique in assessing the kinetics and affinities of protein–protein interactions. Between different ligand–analyte pairs (Figure 3 vs. Figure 4), however, the overall shapes of corresponding sensorgram curves appeared to be markedly different, suggesting distinct and analyte-specific interactions with a particular ligand even under identical experimental conditions. Analysis of the sensorgrams using the 1:1 interaction algorithm for a single exponential interaction gave rise to an estimated *k_on_, k_off_*, as well as *K*_D_ for each representative experiment (Figures 3 and 4). The almost 100-fold difference between the off rate (*k_off_*) of TLR4 and RAGE analyte may underlie the distinct sensorgram curves for pCTS-L interaction with TLR4 (Figure 3) and RAGE (Figure 4), respectively.

## Validation of protocol

Because of our limited access to the Biacore equipment, we did not perform head-to-head comparison between OpenSPR and Biacore for pCTS-L interaction with TLR4 or RAGE. However, we did use both Biacore and OpenSPR to parallelly measure the *K*_D_ between two different interactants (human dermcidin and the extracellular domain of human EGFR) (Qiang et al., 2022). For the traditional Biacore, dermcidin was immobilized on a CM5 chip as a ligand, and recombinant EGFR was applied at five different concentrations using a Biacore T200 instrument (GE Healthcare). The *K*_D_ was determined using the Biacore evaluation software 2.0 supposing a 1:1 binding ratio. For the Nicoya OpenSPR, recombinant dermcidin with a 6× His tag was similarly immobilized on NTA Sensor Chips as a ligand, and recombinant EGFR was applied at three different concentrations to estimate the *K*_D_ using the Trace Drawer Kinetic Data Analysis v.1.6.1. Impressively, the OpenSPR estimated a *K*_D_ of 58.1 ± 29.6 nM (mean ± SEM, n = 3 technical replicates) for dermcidin–EGFR interaction, which was almost identical to the *K*_D_ value (58.8 nM) estimated from using the traditional Biacore SPR technique (Qiang et al., 2022). These findings validate the reliability of using OpenSPR in assessing binding affinities between two different proteins.

When highly purified human pCTS-L was immobilized onto a NTA Sensor Chip and human TLR4 or RAGE were respectively applied as analytes at various concentrations, OpenSPR revealed similarly high affinities of pCTS-L to both TLR4 and RAGE with an estimated *K*_D_ of 20.2 ± 3.5 nM and 3.5 ± 2.6 nM, respectively (Zhu et al., 2023). The calculated *K*_D_ for eight independent experiments is summarized in Table 1 , along with the mean and standard error of mean (SEM), which is indicative of the precision of OpenSPR for estimating a mean *K*_D_. When the SEM was expressed as a fraction of the estimated mean, the resultant Relative Standard Error (RSE) was relatively small (< 20%) particularly for pCTS-L/TLR4 interaction, validating the reliability of using OpenSPR to estimate some protein–protein binding affinities.

**Table 1.** Mean *K*_D_ of eight OpenSPR experiments for pCTS-L interaction with TLR4 or RAGE

**Experiment** #	***K*_D_for pCTS-L/TLR4 (nM)**	***K*_D_for pCTS-L/RAGE (nM)**
1	24.1	2.89
2	20.6	2.66
3	24.9	0.59
4	19.7	0.0023
5	7.7	0.222
6	10.3	0.014
7	15.4	0.019
8	39.0	21.5
**Mean ± SEM**	**20.2 ± 3.5**	**3.5 ± 2.6**
**Relative Standard Error (RSE)**	**17.3%**	

Given the 1:1 stoichiometry of pCTS-L–TLR4 interaction in the ClusPro protein docking (Zhu et al., 2023), we used the 1:1 fitting model for our data analysis. The somewhat imperfect fit between the 1:1 binding model and corresponding data for pCTS-L–TLR4 interaction (Figure 3) did slightly damper our confidence about the *K*_D_ calculated from the *k_off_/k_ on _* of the kinetic data. After all, the 1:1 fitting model cannot handle many artifact responses such as: 1) the bulk shift (bulk refractive index change) resulting from refractive index difference between analyte and running buffer; and 2) the mass transport effect resulting from the transport of analyte from its bulk solution to the Sensor Chip surface. However, we did use identical analyte and running buffer (1× PBS) to eliminate any possible bulk shift or bulk refractive index changes. Meanwhile, the observed curvature of the sensorgram during the association phase of pCTS-L–TLR4 interaction indicated a typical exponential increase of signal that argued against potential mass transport effect (Figure 3). Nevertheless, it remains elusive whether TLR4 analyte heterogeneity (e.g., oligomerization) or minute amounts of contaminant-associated non-specific binding somewhat contribute to the irregularity of pCTS-L–TLR4 sensorgrams (Figure 3).

In the absence of a perfect fit to the simplest 1:1 binding model, the equilibrium constant (*K*_D_) should also be estimated independently by equilibrium analysis if the steady-state binding data covers a wide range of analyte concentrations. Accordingly, we estimated the *K*_D_ for pCTS-L–TLR4 interaction by evaluating the extrapolated steady-state signals as a function of analyte concentrations, assuming that a 10%, 50%, and 90% saturation is obtained at analyte concentrations of 0.1-fold, 1.0-fold, and 10.0-fold of the *K*_D_, respectively. Our estimated *K*_D_ value (< 28.3 nM) from the dose-dependent signal saturation closely agreed with the *K*_D_(20.2 ± 3.5,Table 1) calculated from the *k_off_/k_on_* of the kinetic data. In addition, we performed additional OpenSPR experiments by reversely conjugating TLR4 onto different Sensor Chips before applying pCTS-L as the analyte at serial dilutions (Zhu et al., 2023). Consequently, we obtained a slightly higher but similar *K*_D_(64.6 nM vs. 20.2 ± 3.5 nM) for pCTS-L–TLR4 interaction from improved sensorgrams that exhibited an almost perfect fit to the 1:1 binding model (Zhu et al., 2023), supporting the usefulness of OpenSPR in estimating the *K*_D_ for protein–protein interactions.

**Figure 5.**
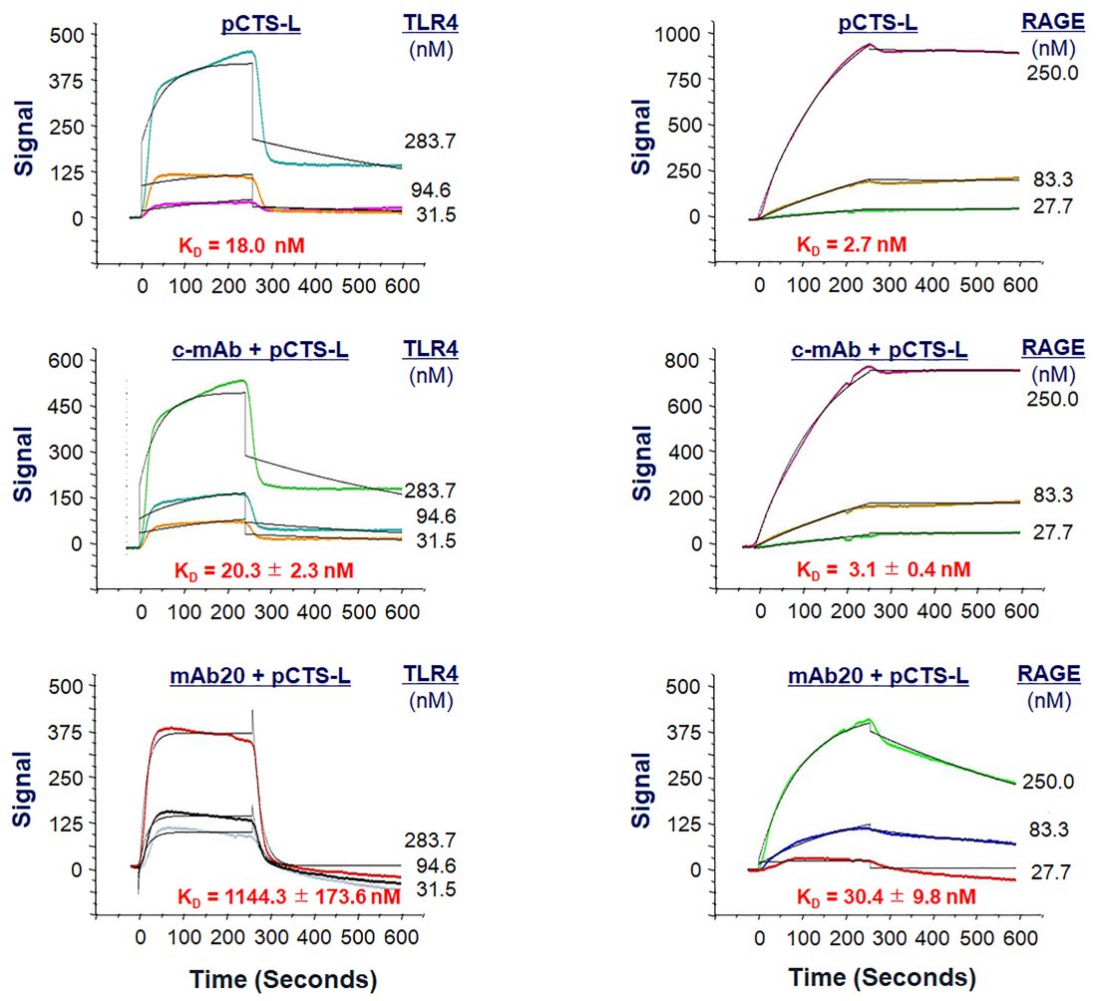
Effect of a pCTS-L-neutralizing monoclonal antibody (mAb20) on pCTS-L ligand interaction with TLR4 or RAGE. Recombinant pCTS-L was immobilized on the NTA Sensor Chip, and an irrelevant control monoclonal antibody (c-mAb) or a pCTS-L–neutralizing mAb (mAb20) was pre-exposed to pCTS-L–conjugated NTA Sensor Chip before subsequent injection of TLR4 (Left Panels) or RAGE (Right Panels) analyte at increasing concentrations to estimate the *K*_D_.

In contrast to typical kinetic analysis performed with alternating cycles of analyte injections and surface regenerations, we employ sequential injections of increasing concentrations of the analyte over the ligand without regeneration between each sample concentration, because harsh regeneration procedures may disrupt ligand capture by the NTA Sensor Chips. However, this single-cycle kinetics OpenSPR gives us an opportunity to assess whether pretreatment with other ligand-binding proteins (e.g., pCTS-L–neutralizing antibodies) affects pCTS-L ligand binding to analytes (e.g., TLR4 or RAGE) subsequently introduced at identical and increasing concentrations. For instance, when an irrelevant control monoclonal antibody (“c-mAb”) was injected onto the pCTS-L–conjugated NTA sensor at an extremely high concentration (e.g.,1200 nM), it did not affect subsequent pCTS-L ligand binding to either TLR4 or RAGE (Figure 5) (Zhu et al., 2023). In sharp contrast, when a pCTS-L–neutralizing IgG (mAb20) was first injected onto the pCTS-L–conjugated NTA sensor, it markedly reduced pCTS-L ligand’s interaction with TLR4 or RAGE analyte subsequently introduced at identical and increasing concentrations, as manifested by an almost 55-fold (from 20.3 ± 2.3 nM to 1144.3 ± 173.6 nM) and 10-fold (from 3.1 ± 0.4 nM to 30.4 ± 9.8 nM) increase in the *K*_D_for TLR4 and RAGE, respectively (Figure 5) (Zhu et al., 2023). The specific impairment of pCTS-L interaction with TLR4 or RAGE by a pCTS-L ligand-specific mAb supports specific interactions between pCTS-L ligand and TLR4 or RAGE analyte.

## General notes and troubleshooting

1. As the OpenSPR uses microfluidic tubing with relatively narrow diameters to save precious analyte samples, all solutions (e.g., running buffers and distilled water) must be filtered through a 0.2 μm filter to prevent any large particulates from entering and clogging the fluidic lines of the OpenSPR. Similarly, analyte solutions should also be carefully inspected for any large particulates before injection into the OpenSPR.
2. In case of tubing clog, the user can purchase a Declogging Kit from the Nicoya Lifesciences to resolve the problem.
3. Injections of different samples should be performed using separate disposable syringes to avoid cross-contamination. If using the glass Hamilton syringe, ensure it is thoroughly washed with running buffer prior to using it for different samples.
4. The minimum sample volume required is the volume of the *Sample Loop* plus 50 μL of excess volume to ensure a uniform sample concentration profile as well as absence of remaining air within the *Sample Loop*.
5. The OpenSPR NTA Sensors provide a convenient capture-coupling technique to immobilize recombinant proteins with 6× His-tags, because its functional NTA groups can capture the protein ligand via its His-tag in the presence of NiCl_2_. These gold nanoparticles conjugated with NTA capture the His-tag ligand in a specific orientation, but the ionic interaction between the His-tag and the Ni-NTA surface is weaker than covalent direct coupling. This may result in some loss of the ligand sample from the surface over time particularly when measuring stronger (low dissociation rate) kinetic interactions with OpenSPR. Because of potential inherent dissociation of the captured ligand, this immobilization method is not recommended for analysis of kinetic systems with slow dissociation.
6. The overall shape of the curve is determined by the analyte concentrations, association rate (*k_on_*), and dissociation rate (*k_off_*) constants, which are independent of the concentrations of analyte and ligand. However, these association and dissociation constants are highly sensitive to the pH and salt concentrations of the solution, reinforcing the importance of keeping experimental conditions constant throughout the studies. Of course, running buffers should not contain chelating or reducing agents (e.g., EGTA) that will remove the necessary Ni^2+^ions or alter the Ni^2+^redox state, both of which may compromise the NTA surface activation.
7. We do not know whether there is a size limit for the ligands that can be used with the OpenSPR, as we have not tried any smaller peptides in the OpenSPR analysis.
8. If the active sites of NTA Sensor Chips were not fully occupied by the ligand, there is a possibility for false positive results if analytes contain multiple surface-exposed histidine residues. Therefore, control experiments using His-tag negative control proteins (with similar molecular weights but unable to interact with the ligand) should be performed to address these potential problems.
